# The efficacy and safety of fire needle for cervical spondylotic radiculopathy: A protocol for systematic review and meta-analysis

**DOI:** 10.1097/MD.0000000000026824

**Published:** 2021-08-06

**Authors:** Kaiyang Xue, Hong Liu, Qiumei Shi, Xianzhu Wang, Yanqin He, Jin Cui, Jing Fu

**Affiliations:** a Guizhou University of Traditional Chinese Medicine, Guizhou, China; b The First Affiliated Hospital of Guizhou University of Traditional Chinese Medicine, Guizhou, China.

**Keywords:** cervical spondylotic radiculopathy, fire needle, meta-analysis, protocol, systematic review

## Abstract

**Background:**

Cervical spondylotic radiculopathy (CSR) is one of the most common types of cervical spondylosis, and its treatments are mainly for relieving radicular pain and improving dysfunction. The existing randomized controlled trials (RCTs) suggest that fire needle may be a potential therapy in the treatment of CSR, but there is no evidence-based medical evidence to date. Therefore, this study will systematically evaluate the efficacy and safety of fire needle in the treatment of CSR.

**Methods:**

*We will search for 7 electronic databases (PubMed, EMBASE, Cochrane library, China National Knowledge Infrastructure, Chinese Scientific Journals Database, Sinomed, and Wanfang Database) and 2* trial registration platforms (ClinicalTrials.gov and Chinese Clinic Trials.gov) *to* collect *eligible studies.* The RCTs related to fire needle for CSR and *published up to June 30, 2021* will be included, regardless of language. We will consider the visual analogue scale as the primary outcome and the secondary outcome will include cervical range of motion, assessment of muscle strength, neck disability *index*, the MOS item short from health survey, activities of daily living, total efficiency, and adverse reactions. We will use the standard proposed in Cochrane Handbook 5.1.0 to assess the *quality and* bias risk of every RCT, and all analyses will be conducted through RevMan software V5.3 (Copenhagen: Nordic Cochrane Center, Cochrane, Collaborative Organization, 2014).

**Results:**

This systematic review and meta-analysis will provide a convincing synthesis of existing evidences on the efficacy and safety of fire needle for CSR, and the results will be submitted to a peer-reviewed journal for publication.

**Conclusion:**

The results of this study will provide high-quality evidence of fire needle in the treatment of CSR for clinical decision-making.

**INPLASY registration number:**

INPLASY202170041.

## 1 Introduction

Cervical spondylotic radiculopathy (CSR) is a syndrome characterized by compression of one or more adjacent nerve roots due to degenerative changes in cervical bone, intervertebral discs, ligaments, facet joints and other accessory tissues.^[1]^ Among them, as many as 80% of patients presented with C6 or C7 root involvement.^[2]^ The occurrence of CSR is mostly associated with the aging of the population, contemporary social pressure, and lifestyle, which performs pain along the cervical nerve roots, dyskinesias, paresthesias, and weakened reflexes.^[3]^ With the progression of the disease, severe cases may have muscle atrophy, or even loss of upper limb function, resulting in a decline in productivity and quality of life.^[4,5]^ The incidence of CSR accounts for about 60% to 70% of all cervical spondylosis, which is the most common type of cervical spondylosis in clinical practice. A recent systematic review of the epidemiology of CSR shows that the prevalence of CSR ranged from 83 to 179 per 100,000 people, and the prevalence of females is higher than that of males.^[6,7]^

Currently, the treatment strategies recommended by the guidelines for CSR contain surgical and conservative treatment, including traction, drugs, massage, acupuncture and moxibustion, and physical therapy, etc.^[8–10]^ The survey shows that the success rate of surgical treatment is between 80% and 95%,^[11]^ among which discectomy and fusion are more commonly used.^[12]^ However, 4% of postoperative patients experienced adverse events such as reduction of intervertebral disc height and degeneration of adjacent segments.^[13]^ In recent years, surgical methods for CRS have been continuously optimized and the number of operations has continued to increase, but the exact indications of surgery and the advantages of surgery compared with conservative treatment have not been definitely verified.^[14]^ More than 75% of patients can significantly improve their condition within 3 months with conservative treatment, which remains the first choice for most patients with CSR.^[15,16]^ Among them, acupuncture and moxibustion therapy including fire needle has become a commonly used clinical therapy due to its outstanding curative effect, non-toxic side effects and economic benefits.

The origin of fire needle can be traced back to the pre-Qin period. It is a kind of external treatment method that uses heated needles to stimulate acupoints or lesions quickly to cure diseases.^[17–19]^ This method combines the mechanical stimulation of acupuncture and the warm stimulation of moxibustion, which can achieve synergistic effects. It has the effects of warming the meridians, dispelling cold and dehumidifying, and invigorating yang, and is often used clinically for nerve damage, motor dysfunction and dermatological diseases. Studies have shown that fire needle can effectively relieve radicular pain,^[20]^ somatosensory disorder,^[21]^ disabled physical activity^[22]^ and other CSR-related symptoms, and its mechanism may be related to the intervention of pain conduction, regulation of inflammatory response and improvement of local blood circulation. Fire needle can down-regulate the levels of pain transmitters such as 5-hydroxytryptamine and substance P in the central and peripheral nervous systems,^[23,24]^ as well as the levels of tumor necrosis factor and interleukin-1 in serum,^[25–27]^ and mobilize the body to release more vascular endothelial growth factor involved in trauma recovery actively to promote the repair of nerve damage or stimulating symptoms.^[28]^

So far, the efficacy and safety of fire needle in the treatment of CSR have not reached a consensus. The aim of this study is to evaluate the efficacy and safety of fire needle for CSR by systematically reviewing and analyzing the currently available randomized controlled trial (RCTs), which will provide evidence support for clinical decision-making.

## 2 Methods

### 2.1 Study registration

The protocol has been registered on the INPLASY platform (Registration Number: INPLASY202170041, https://inplasy.com/inplasy-2021-7-0041). We will report this protocol according to the preferred reporting items for systematic reviews and meta-analysis protocols statement guidelines.^[29]^

### 2.2 Inclusion and exclusion criteria

#### 2.2.1 Type of study

We will include the RCTs which evaluating the efficacy and safety of fire needle for CSR in any language, whether published or not. Others such as randomized crossover trials, animal trials, medical cases, and only published in the form of abstracts will be excluded.

#### 2.2.2 Participants

We will include participants who are clearly diagnosed with CSR according to any recognized diagnostic criteria, without restrictions on age, gender, profession, ethnicity, and source of cases.

#### 2.2.3 Interventions and controls

Two types of RCTs will be included:

- The experimental group is treated with fire needle alone, while the control group accepts other therapies.
- The experimental group is treated with fire needle combined with other therapies, while the control group accepts the same other therapies.

#### 2.2.4 Outcomes

##### 2.2.4.1 Primary outcomes

The primary outcome is visual analogue scale.

##### 2.2.4.2 Secondary outcomes

The secondary outcomes are cervical range of motion, assessment of muscle strength, neck disability index, the MOS item short from health survey, activities of daily living, total efficiency, and adverse reactions.

### 2.3 Data sources and search strategy

The following 7 electronic databases and 2 trial registration platforms will be searched to collect eligible studies published up to June 30, 2021: PubMed, EMBASE, Cochrane library, China National Knowledge Infrastructure, Chinese Scientific Journals Database, Sinomed, Wanfang Database, ClinicalTrials.gov and Chinese Clinic Trials.gov. We will use various combination of medical subject headings and free words to search for terms such as “cervical spondylotic radiculopathy”, “ cervical radiculopathy ”, and “fire needle”, a search strategy in PubMed will be demonstrated in Table 1.

**Table 1:** Search strategy in PubMed.

No.	Search terms
1	Cervical spondylotic radiculopathy [mh]
2	Cervical spondylotic radiculopathy [tw]
3	Cervical spondylosis [tw]
4	Cervical radiculopathy [tw]
5	Cervical syndrome [tw]
6	Nerve root cervical spondylosis [tw]
7	Cervical spine radiculopathy [tw]
8	#1 or #2 or #3 or #4 or #5 or #6 or #7
9	Fire needle [tw]
10	Fire needle therapy [tw]
11	#9 or #10
12	Animals [mh]
13	Humans [mh]
14	#8 and #11
15	#12 not #13
16	#14 not #15

### 2.4 Data collection and analysis

#### 2.4.1 Study selection

After using Endnote X9 (Clarivate Analytics US LLC) to exclude repetitive literatures, 2 researchers will independently read the title and abstract, conduct a preliminary screening based on the inclusion criteria, and then check the full text to determine whether it will be included. If there is any disagreement, further discussion and verification will be conducted, or the third researcher will assist in the resolution.

**Figure 1:**
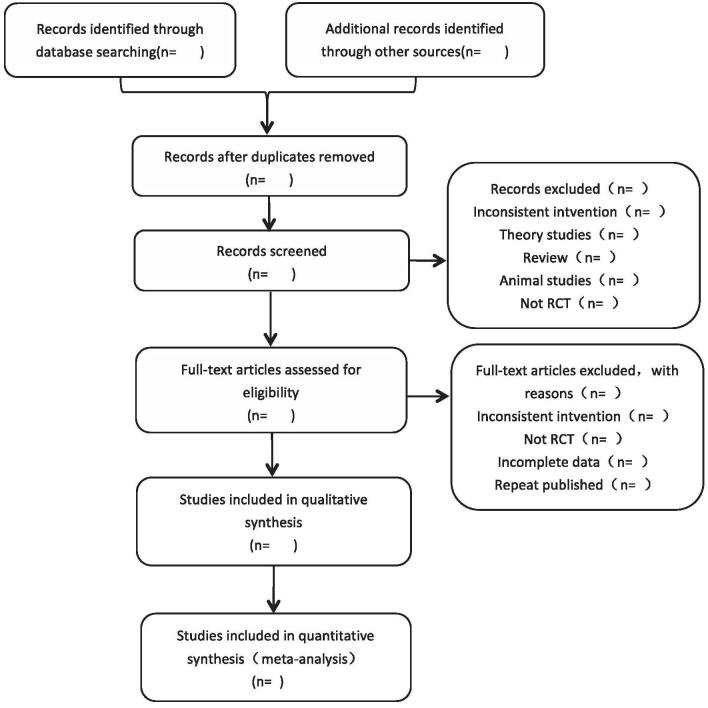
PRISMA-style flow chart of literature screening. PRISMA = preferred reporting items for systematic reviews and meta-analysis.

The details of screening process will be shown in the preferred reporting items for systematic reviews and meta-analysis flow chart (Fig. 1).

#### 2.4.2 Data extraction

Two researchers will independently complete the data extraction work through a table containing the following information: publishing features (title, first author, year of publication, institutional units, journals), participants and interventions (baseline data, randomization and blinding, treatment site, adverse events, follow-up time), outcomes data. The data will be cross-checked after extraction and any disputes will be resolved by discussion with a third researcher.

#### 2.4.3 Risk of bias assessment

This study will use the bias risk assessment tool in the Cochrane Handbook 5.1.0 to assess the risk of bias.^[30]^ The content includes 7 items, such as random sequence generation method, allocation concealment method, blinding of clinicians and patients, blinding of outcome evaluators, data integrity, selective reporting, and other sources of bias. According to the literature information, the risk of bias will be judged to be low, high, and unclear. Two researchers will assess the risk of bias repeatedly and independently, then cross-check the results. Any differences will be determined by consulting a third party.

#### 2.4.4 Managing missing data

If there is data missing in the literature, try to contact the original author through e-mail or telephone, and get the relevant information needed. If it is not available, it will be explained in the article.

#### 2.4.5 Data synthesis

The Revman 5.3 statistical software provided by the Cochrane Collaborative network will be used to summarize and analyze the data included in the literature. Mean difference will be used as the statistic of continuous data. Relative risk will be used as the statistic of the dichotomous data. Both continuous data and dichotomous data are expressed in terms of effect size and its 95% confidence interval.

#### 2.4.6 Assessment of heterogeneity

In this study, the Cochran Q test (qualitative) and I^2^ statistical test (quantitative) will be used to test the heterogeneity of the results of each study. When there are no dominant heterogeneity (*P* > .1, I^2^ < 50%), the fixed effects model will be used for analysis. When there are significant heterogeneity in the results (*P* < .1, I^2^ *>* 50%), the random effects model will be used for analysis, and the sensitivity analysis or subgroup analysis will be conducted to identify possible sources of heterogeneity.

#### 2.4.7 Subgroup analysis

Based on the clinical experience in the application of fire needle, when there is significant heterogeneity in the analysis results, we will conduct subgroup analysis according to the basic characteristics of the patients and the treatment course to explore the potential sources of heterogeneity.

#### 2.4.8 Sensitivity analysis

In order to verify the robustness of the meta- analysis results, we will remove the included literatures in order of quality from low to high, and recombine the effect sizes to compare with the previous analysis results. If there is no significant change in the results of the combination, the results of the meta-analysis are reliable. If the results of the combination change significantly, the sensitivity is high and the results are not reliable.

#### 2.4.9 Assessment of publication bias

We will draw a funnel plot to qualitatively measure whether there is potential publication bias in this study, and use Egger test to determine whether the funnel plot is symmetrical. If the funnel plot shows high symmetry and *P* > .05, it can be considered that there is no publication bias. If the symmetry of distribution morphology is poor, or *P* < .05, it is considered that there is publication bias, and further screening of literatures is needed.

#### 2.4.10 Assessment of the quality of evidence

We will assess the quality of evidence for each research according to the Grading of Recommendations Assessment, Development and Evaluation working group approach, and divide the evidence into high, moderate, low, and very low.

### 2.5 Ethics and dissemination

This study only extracts and uses data from the existing literature and does not involve patients or public participation, so ethical approval is not required. Our goal is to submit the results of this systematic review to peer-reviewed journals for publication.

## 3 Discussion

Fire needle therapy combines the characteristics of “acupuncture” and “moxibustion”, and is a characteristic Chinese medicine therapy with great potential for development. However, due to insufficient evidence of clinical efficacy, the clinical application of fire needle is more limited than milli-needle, moxibustion, massage, and other therapies. Based on literature analysis and clinical experience, we believe that fire needle therapy can treat CSR by reducing nerve compression and repairing nerve damage. The current systematic reviews for the treatment of CSR mostly focus on acupuncture and moxibustion, but there is a lack of systematic review and meta-analysis on the use of fire needle alone in the treatment of CSR. A number of RCTs of fire needle in the treatment of CSR have shown that fire needle can alleviate the symptoms of CSR, but the sample sizes of these studies are small, and there are differences in clinical efficacy. We think it is necessary to use comprehensive retrieval methods to evaluate the efficacy and safety of fire needle in the treatment of CSR.

In this systematic review, we will conduct research in strict accordance with the agreement and try our best to achieve the following points: comprehensive and accurate literature retrieval, strict literature screening, standard bias risk assessment, scientific data analysis, and objective quality assessment. We believe that the results of this systematic review can provide high-quality evidence for clinicians to make decisions and help enhance the quality of life of CSR patients.

## Author contributions

**Conceptualization:** Jing Fu.

**Funding acquisition:** Jin Cui.

**Investigation:** Qiumei Shi, Xianzhu Wang, Yanqin He.

**Methodology:** Kaiyang Xue, Hong Liu.

**Writing** – **original draft**: Kaiyang Xue, Hong Liu, Jing Fu.

**Writing** – **review & editing:** Jing Fu, Jin Cui.
